# Understanding Sperm Quality for Improved Reproductive Performance

**DOI:** 10.3390/biology12070980

**Published:** 2023-07-10

**Authors:** Pilar Santolaria, Jessica P. Rickard, Rosaura Pérez-Pe

**Affiliations:** 1 Grupo BIOFITER, Departamento Producción Animal y Ciencia de los Alimentos, Instituto Universitario en Ciencias Ambientales de Aragón (IUCA), Escuela Politécnica Superior, Universidad de Zaragoza, 22071 Huesca, Spain; 2 RMC Gunn Building, School of Life and Environmental Sciences, Faculty of Science, The University of Sydney, Sydney, NSW 2006, Australia; jessica.rickard@sydney.edu.au; 3 Grupo BIOFITER, Departamento Bioquímica y Biología Molecular y Celular, Instituto Universitario en Ciencias Ambientales de Aragón (IUCA), Facultad de Veterinaria, Universidad de Zaragoza Miguel Servet, 50013 Zaragoza, Spain; rosperez@unizar.es

The assessment of semen quality is used to identify factors that influence sperm performance and diagnose male infertility. From the earliest works to the present day, a considerable number of analytical techniques have been developed. These tests study many aspects of the morphology and physiology of the spermatozoan; however, their ability to predict male fertility remains low. Some of these techniques have been automated, which can make the test results more objective, but others still require subjective evaluation. The introduction of computer-assisted sperm analysis (CASA) systems and flow cytometry has revolutionized sperm quality analysis in recent decades. However, the use of these advanced techniques remains experimental, with only a few of them having successfully led to practical applications in routine commercial semen evaluation. Future sperm biology research should focus on developing analytical techniques that have a greater capacity to predict male fertility and can be used in both the laboratory and the field. Field adaptation will require progress in automation and simplification, to produce precise, economical and efficient techniques. Nevertheless, as technology advances in these analytical tests and research continues, our greater understanding of male fertility will aid the development of new methods of sperm evaluation.

This Special Issue of Biology entitled “Sperm quality: Past, present and the future knowledge we need” focuses on understanding the quality of spermatozoa in three sub-sections: (1) the function of the sperm cell, (2) its ability to withstand cryopreservation and (3) its performance both in vitro and in vivo. Combined, this Special Issue contains 11 published peer-reviewed papers.

## 1. Function of the Sperm Cell

An important endeavor in the creation of this Special Issue was to delve into aspects related to the biology and physiology of spermatozoa. In this regard, sperm capacitation is essential for the acquisition of fertilizing capacity, and, although it has been extensively studied in many species, there are still aspects that remain unclear. Many studies have been conducted to establish a suitable capacitation medium for different species, because effective in vitro sperm capacitation is required for successful in vitro fertilization (IVF). Most of the media examined include bovine serum albumin (BSA) and bicarbonate, but these compounds may not be necessary to capacitate sperm from all species. Chaves et al. [1] demonstrated that BSA is crucial for pig sperm to elicit in vitro capacitation and trigger subsequent progesterone-induced acrosome exocytosis. In contrast, although exogenous bicarbonate does not appear to be indispensable, it shortens the time needed to reach that capacitated status. The authors of this work concluded that media containing BSA and low levels of or no bicarbonate were the most suitable for inducing the capacitation of pig sperm, maintaining higher motility and plasma membrane integrity than those with high bicarbonate levels. These results do highlight the existence of species-specific mechanisms that are regulating the successful performance of sperm capacitation in vitro.

The development and maturation of spermatozoa, as well the acquisition of motility and capacitation, are strongly coordinated by sperm protein phosphorylation, among other protein post-translational modifications. Serrano et al. [2] reviewed and summarized the current knowledge of protein phosphorylation in human spermatozoa as the mechanism responsible for the regulation of processes necessary to achieve successful fertilization. The application of global phosphoproteomic profiling technology in evaluating sperm quality would enable the identification of male infertility biomarkers and could reveal insights into cases of idiopathic infertility in humans. In this regard, many couples attending infertility clinics are eventually enrolled in an intracytoplasmic sperm injection (ICSI) cycle after several failed attempts of pregnancy. In this procedure, the selection of the most adequate sperm to be injected inside the egg is crucial to the cycle’s success. Magnetic activated cell sorting (MACS) is a technique that removes sperm that have begun undergoing an apoptotic process from a semen sample. This technique is not routinely performed prior to ICSI, but is usually offered as an option to increase the chances of success. In the retrospective, multicenter, observational study conducted by Gil-Juliá et al. [3], the researchers assessed the impact of MACS on reproductive outcomes. Their results suggested that non-apoptotic sperm selection via MACS prior to ICSI with autologous oocytes reduces the number of embryos required to be transferred in order to obtain a live birth, but the method has limited clinical impacts. Consequently, MACS should not be recommended to all infertile couples before performing an ICSI cycle. Interestingly, this technique in combination with the use of antibodies has been shown to be effective for sperm sexing. In the dairy industry, the use of sexed sperm to increase the percentage of female calves in offspring has been long considered. The technique currently used for this purpose is flow cytometry combined with a cell sorter which separates X-chromosome-bearing sperm (X-sperm) and Y-chromosome-bearing sperm (Y-sperm) based on the difference in their DNA content. Immunoseparation is presented as a cheaper and less stressful alternative for spermatozoa sexing by Sringarm et al. [4], who showed that the use of magnetic microbeads coupled with scFv antibodies against Y-sperm (PY-microbeads) produced 82.65% of X-chromosome sperm in the selected fraction with acceptable sperm quality.

## 2. Impact of Cryopreservation on Sperm Quality

The use of sperm preservation has long been considered a vital reproductive technology in human medicine, livestock artificial breeding and wildlife conservation programs. Cryopreservation, in particular, not only facilitates biobanks and the storage of precious genetic material but also maximizes long-term fertility and genetic gain. Ever since glycerol was discovered to be an effective cryoprotectant in 1947, the species-specific protocols and media used have undergone extensive research to optimize sperm survival and quality post-thaw. The basic principles involved in the freeze–thaw process, including the cooling curve, freezing rate and thawing, will always remain; however, as technology advances and procedures are automated, there is always the opportunity to update and improve the quality of spermatozoa post-thaw. Within this Special Issue, knowledge on the impact of cryopreservation on the metabolome of turkey spermatozoa was reported, recommendations were made regarding the use of the sperm-rich fraction or entire ejaculate for improved boar sperm storage and fertility, and a novel enzyme was suggested to reduce the viscosity associated with wildlife ejaculates, improving the cryosurvival and availability of rhinoceros spermatozoa for wildlife conservation programs. These scientific papers represent some of the compelling research being conducted to advance our knowledge and understanding of sperm quality post-cryopreservation.

Of all the animal industries, research on the impact of cryopreservation on avian sperm quality is likely to be the most limited in comparison to that of cattle, pigs or sheep. Paventi et al. [5] used nuclear magnetic resonance (NMR) to create a profile of turkey spermatozoa both prior to and after freezing. They observed a positive correlation between amino acid levels with physiological changes in sperm parameters, with the exception of glycine, which was revealed to have a negative association. Interestingly, glycine was also seen to increase following cryopreservation. From these results, not only was a metabolomic profile of turkey spermatozoa created, but authors also identified biomarkers for sperm freezability and quality. Furthermore, they identified potential strategies through the supplementation of metabolites which could be used to improve the success of turkey sperm cryopreservation.

Boar sperm storage protocols (both liquid and frozen) have undergone extensive development and optimization over time. A recent objective was to maximize the number of doses produced for artificial insemination from one ejaculate per male. Luongo et al. [6] investigated the impact of different boar ejaculate fractions on the quality of sperm liquid stored from the sperm-rich fraction. There was no impact of incubating the entire ejaculate or mixing the sperm-rich and intermediate fraction on sperm quality, pregnancy following artificial insemination or offspring survival. This suggests the possibility that boar ejaculates could be used more effectively for both sperm storage and insemination protocols, increasing the reproductive and economic efficiency of artificial reproductive technologies for the pig industry.

Ejaculates collected from wildlife species via electro-ejaculation are often highly viscous. It is suggested that this method of semen collection, artificially stimulates the accessory sex glands, altering the contribution of seminal plasma and physiological parameters of the ejaculate. Like that of the boar, the ejaculate of the rhinoceros is also fractionated, with a sperm-rich fraction predominantly being used for cryopreservation and artificial insemination. However, this renders the very expensive and labor-intensive procedure of collecting semen very inefficient, especially when considering the risk posed to valuable endangered animals. Any semen obtained from these viscous fractions are often of poor quality and do not survive the freezing process. Rickard et al. [7] investigated the use of the enzyme papain, which has successfully been used in alpaca freezing protocols, to reduce the viscosity of rhinoceros ejaculates. Here, they reported that papain not only increased the quantity of spermatozoa available for use from one ejaculate but also improved the motility and kinematics of spermatozoa post-thaw compared to those of the sperm-rich fraction, without detriments to viability, DNA integrity or acrosome integrity. This work substantially increases our knowledge of rhinoceros sperm quality during cryopreservation, the reduced viscosity of samples also enables the use of advanced semen assessment technology such as CASA and flow cytometry. It also raises the possibility of improving the ejaculate quality collected of other wildlife species for conservation and breeding purposes.

## 3. Relationship between Sperm Quality and Fertility

There is a direct relationship between sperm quality and fertility. Sperm quality refers to the health and characteristics of sperm cells, including their count, motility, morphology, and viability. These factors are crucial for successful the fertilization of an egg and the establishment of a viable pregnancy. Evaluating and addressing any issues with sperm quality through proper diagnosis and appropriate treatments can significantly improve the chance of inducing pregnancy. However, achieving satisfactory predictions based on the in vitro evaluation of sperm quality remains a challenge. Overall, the combination of scientific discoveries, technological innovations, advancements in optical physics, and computing techniques has significantly advanced our understanding of semen quality and its impact on fertility. These advancements could improve, significantly, the accuracy of semen analysis.

In this sense, testicular ultrasound, a non-invasive diagnostic procedure, could be a useful tool. Recent ultrasound-video analysis and software developments have allowed the visualization of tissue at the microscopic level. Carvajal-Serna et al. [8] revealed that echotexture analysis via ultrasound video could be a valuable tool for assessing the breeding soundness of rams. An increase in the number of white and grey pixels could indicate a decrease in seminal quality, and tubular density and lumen area could be predictors of good seminal quality. Therefore, ultrasound video analysis could be a useful tool for evaluating the fertility of rams, either for storage, artificial insemination or natural joining on the farm.

On the other hand, the analysis of sperm performance under in vitro conditions provides a good indication of fertilizing potential. Parameters such as motility, swimming kinetics, acrosome integrity, and ATP content are thus examined in efforts to characterize such potential. Hamster species are a good model for studying sperm parameters that are key determinants of fertilizing capacity because these species are at the higher end of the diversity of mammalian sperm morphology and performance. In vitro functional studies demand that sperm remain viable during a long period of time under conditions that resemble those in the female tract. Sperm from certain species require a supplementation of the incubation medium with factors that stimulate viability and motility, or that promote the acquisition of fertilizing capacity. Molecules important for sperm performance in hamsters have been identified, namely D-penicillamine, hypotaurine and epinephrine (PHE). Tourmente et al. [9] investigated the effect of PHE on spermatozoa from five hamster species incubated for up to 4 h, revealing that supplementation with a combination of D-penicillamine, hypotaurine and epinephrine maintains or improves the performance of spermatozoa from five hamster species in different manners, depending on the species.

Regarding the relationship between the quality and fertility parameters, Yániz et al. [10] investigated whether or not differences in bull fertility are associated with variations of sperm quality. Differences between high- and low-fertility bulls were found mainly in parameters related to sperm acrosome integrity when using a new fluorescence method that allowed the clear and precise detection of the sperm plasma membrane and acrosome: the ISAS3Fun method. It was concluded that the simultaneous assessment of sperm viability and acrosome integrity with the ISAS3Fun method is accurate and seems to have greater potential in discriminating between high- and low-fertility bulls than do more conventional in vitro sperm quality tests. These results may help to predict the breeding soundness of bulls used in artificial insemination, which is important for the dairy industry.

Finally, swine reproduction efficiency is determined by the fertility potential of the sow and sperm quality. The objective of Barquero et al.’s study [11] was to compare boar sperm motility and kinematic features to evaluate their relationship with reproductive success after artificial insemination. The movement patterns of boar ejaculates were analyzed using a computer-assisted semen analysis (CASA)-Mot system, and the kinematic values of ejaculate clusters were assessed. They showed that kinematic analysis of boar ejaculates reveals kinematically separate populations. There were also differences between the sperm kinematic variables in terms of sire line. However, there was no overall significant difference between dam lines assessed via multivariate procedures. The fertility variables characterized according to the sire genetic line did not show differences, except for the significantly fewer stillbirths in Pietrain boars. Sperm kinematic variables may provide the capacity to predict litter size variables, albeit a limited one. Nevertheless, the analysis of ejaculates, organizing them into clusters, did not provide the capacity to predict litter size variables.
